# Teachers’ labeling of student behavior problems: a multiperspective study of teacher, student, and classroom conditions

**DOI:** 10.3389/fpsyg.2026.1704331

**Published:** 2026-04-10

**Authors:** Boris Eckstein, Urs Grob, Kurt Reusser, Alexander Wettstein

**Affiliations:** 1 Department of Research and Development, Centre of Inclusion and Health in Schools, Zurich University of Teacher Education, Zurich, Switzerland; 2 Faculty of Arts and Social Sciences, Institute of Education, University of Zurich, Zurich, Switzerland; 3 Department of Research and Development, University of Teacher Education Bern, Bern, Switzerland

**Keywords:** behavior problems, interactionism, labeling approach, multi-informant design, perception bias, structural equation modeling

## Abstract

Many teachers are concerned that students with behavior problems may strain teaching, classmates, or themselves. Although these concerns are understandable, research has yet to clarify the extent to which the teacher-assigned label “behavior problems” is substantiated by students’ actual behaviors – and the extent to which it is due to other conditions biasing teachers’ perceptions. Addressing this research gap, the present paper explores the conditions of teachers’ labeling tendencies in a multi-informant, multilevel survey study. A total of 85 elementary school teachers and 1,412 students answered a questionnaire. Teachers reported the degree to which they consider each student in their class to have behavior problems (labeling tendency). Four randomly assigned classmates rated the frequency of the students’ undisciplined behaviors as a presumed condition of teachers’ labeling tendencies. Further, we assessed non-behavioral student characteristics, teacher characteristics, and contextual factors as additional labeling conditions. A two-level structural equation model yielded significant effects for the individual students’ indiscipline frequency, sex, and subjective experience of instructional clarity on the teachers’ tendencies to label them (level 1). At level 2, the teachers’ general sensitivity to disturbances and work-related stress experience were found to be significant conditions of their general labeling tendency across students. No significant effects were found for students’ collective level of indiscipline or their average experience of instructional clarity in the classes. In sum, the effects explain 54% of the variance in teachers’ tendency to label individual students as having behavior problems (level 1) and 37% of the variance in their general labeling tendency across students (level 2). The main findings indicate that the individual students’ behaviors largely substantiated the teachers’ labeling tendencies (large effect size) – the additional labeling conditions that had little or nothing to do with the students’ behavior imply perception biases (small to medium effect sizes).

## 1 Introduction

Student behavior problems are considered one of the most crucial challenges for schools (de Boer et al., 2011; MacFarlane and Woolfson, 2013). Many teachers find them distracting or annoying – for classmates and for themselves (Eckstein, 2019). Research shows that student behavior problems can even pose a risk factor for teacher health (Wettstein et al., 2021; La Marca et al., 2023). These issues are also worrying the general population (Schwab, 2015). Such concerns are understandable when it comes to severe forms of behavior problems like bullying or physical violence.

However, the term “behavior problems” is imprecise. First, it denotes various forms, including innocuous variants like daydreaming that hardly disturb teachers or classmates (Arbuckle and Little, 2004). Beyond that, severe behavior problems (e.g., violence) occur comparatively rarely in schools (Crawshaw, 2015). Moreover, recent studies suggest that teachers have difficulty accurately appraising the frequency of student behavior problems (Eckstein, 2018; Wettstein et al., 2023).

In other words, although public discourse on student behavior problems insinuates that the concept is unambiguously clear, it is in fact vague. This vagueness is concerning because the label “behavior problems” poses a risk to students’ development (Bernburg, 2019) and is a source of controversy in educational policy (Bernhardt, 2007). This gives rise to a research desideratum we address in this article: studies should investigate the extent to which the label “behavior problems” is substantiated by students’ behaviors and the extent to which it is due to other, idiosyncratic conditions.

## 2 Conceptualizing student behavior problems

The literature addresses various forms of student behavior problems (Stein, 2014). These include academic disengagement (e.g., daydreaming, silent off-task behavior), indiscipline (e.g., talking out of turn, walking around without permission), and aggression (e.g., humiliation, mobbing, physical violence). All forms have in common that they deviate from social norms, such as classroom-specific communication rules or general socio-moral norms like respect (Eckstein, 2018). Deviant student behaviors can disrupt the teaching-learning processes and emotionally burden teachers or classmates (Thiel, 2016). In this sense, the term “behavior problems” is often used to describe activities with interpersonal effects (“shows behavior problems”). However, research found interindividual differences in teachers’ and students’ experience of deviant behaviors – not everyone considers the same behaviors equally troublesome (Eckstein, 2019). For example, when a student calls out a response in a class discussion without being prompted by the teacher, there may be differing opinions on whether this behavior is inappropriately impulsive or vividly engaged.

Beyond that, the term “behavior problems” is also used to describe intrapersonal student characteristics (“has behavior problems”). These can be differentiated by the degree of severity: mental disorders requiring treatment (e.g., conduct disorder), border cases on the threshold of a disorder (e.g., moderate impulsivity), and mild cases (e.g., occasional fidgetiness) (Schwenck, 2012). Epidemiologic research indicates that 6%–10% of all children and adolescents suffer from mental disorders, and a further 10%–13% are border cases, with prevalence rates increasing with age (Ihle and Esser, 2002; Myschker and Stein, 2014). This research provided important insights into students’ mental health and disorders at a general level. However, its findings raise several questions. First, the statistics refer to all mental disorders, including those with a shallow potential to disturb teachers or classmates (e.g., depression). Furthermore, the prevalence rates reported across studies vary considerably, partly due to methodological issues (Korsch and Petermann, 2012). Finally, it is unknown how many students with mental problems attend regular or special schools.

Further evidence of the problem’s extent is provided by research on the frequency of deviant student behaviors in the classroom. Studies have repeatedly shown that academic disengagement represents the most frequent form, followed by indiscipline, while aggression has the lowest incidence (Harrison et al., 2012; Munn et al., 2013; Oertel et al., 2015). Interestingly, most teachers and students consider indiscipline the most troubling form of student behavior problems, not the more severe forms such as violence (Little, 2005). This is because undisciplined behaviors can become cumulatively stressful when occurring frequently, even though single incidents may seem trivial. Therefore, the frequency with which students show indiscipline likely plays a central role in teachers’ appraisal of behavioral problems.

Given these initial spotlights from the literature, it can be stated that the term “behavior problems” refers to a perspective-dependent phenomenon of various forms and degrees. Due to this complexity, there is no universally accepted definition (Fröhlich-Gildhoff, 2007). To achieve a better understanding, it is essential to examine not only the behavior itself but also how the social environment perceives it. This paper addresses teachers’ perspectives on student behavior problems.

### 2.1 Academic traditions emphasizing different problem aspects

The discourse on student behavior problems is strongly influenced by pathogenetic approaches that relate the phenomenon primarily to the behavior itself and its causes (e.g., Petermann and Natzke, 2008). This includes student characteristics (e.g., ADHD), family problems (e.g., parenting deficits), or contextual factors (e.g., poor classroom management). Empirical studies in this tradition are important for understanding how student behavior problems arise. However, since the phenomenon is perspective-dependent, this research faces conceptual and methodological challenges (Eckstein, 2018). The varying prevalence rates cited above can be seen as a symptom of uneven rigor in this respect. To overcome these issues more effectively, future studies need to take into account who considers which behaviors problematic in which situations, and why.

The psychology of social perception provides a valuable approach to investigating how teachers and classmates perceive, interpret, and evaluate student behavior (e.g., Hall et al., 2016). Research in this tradition found several mechanisms that may bias teachers’ perspectives: the halo effect (Höhn, 1967/1980), the primacy effect (Timmermans et al., 2021), the impact of social categories on the perception of individuals (Tajfel et al., 1971), and selective attention (Simons and Chabris, 1999). In sum, teachers’ appraisals of student behavior problems reflect objective factors (i.e., the students’ actual behavior) and idiosyncratic conditions (Lazarus and Folkman, 1984).

The labeling approach is helpful for understanding how social norms are established in the classroom and why teachers might assign the label “behavior problems” to students (e.g., Goffman, 1963; Blumer, 1969). This tradition is based on symbolic interactionism, according to which people attribute meaning to other people, objects, or behaviors – and act because of these attributions (Mead, 1934). A distinction can be made between formal labeling (e.g., official diagnosis of special educational needs) and informal labeling (e.g., personal opinion) (Rocheleau and Chavez, 2015). Informal labeling can be considered an early step in deviant careers (Becker, 1963): when a teacher considers a particular student to have behavior problems, he/she will likely monitor this student with heightened alertness. As a consequence, the teacher may overestimate the problem’s extent and, thus, sanction the student comparatively harshly (Hofer, 1986). As a defense reaction, the student may respond with even more problematic behaviors (self-fulfilling prophecy). This article addresses teachers’ informal labeling without repeating the prefix “informal” whenever it is mentioned.

### 2.2 Teacher-perceived student behavior problems – an interactionist concept

We understand teachers’ perception and labeling of student behavior problems as specific processes in the context of pedagogical interactions. This concept relies on an interactionist framework that integrates arguments from pathogenetic approaches, psychology of social perception, and labeling theory (Eckstein et al., under review): pedagogical interactions comprise the reciprocally interrelated behaviors of teachers and students as well as their subjective experiences during the interaction. Furthermore, these interactional processes are influenced by the actors’ personal characteristics and contextual factors (Pianta, 1999; Wubbels et al., 2015; Schweer et al., 2017).

**FIGURE 1:**
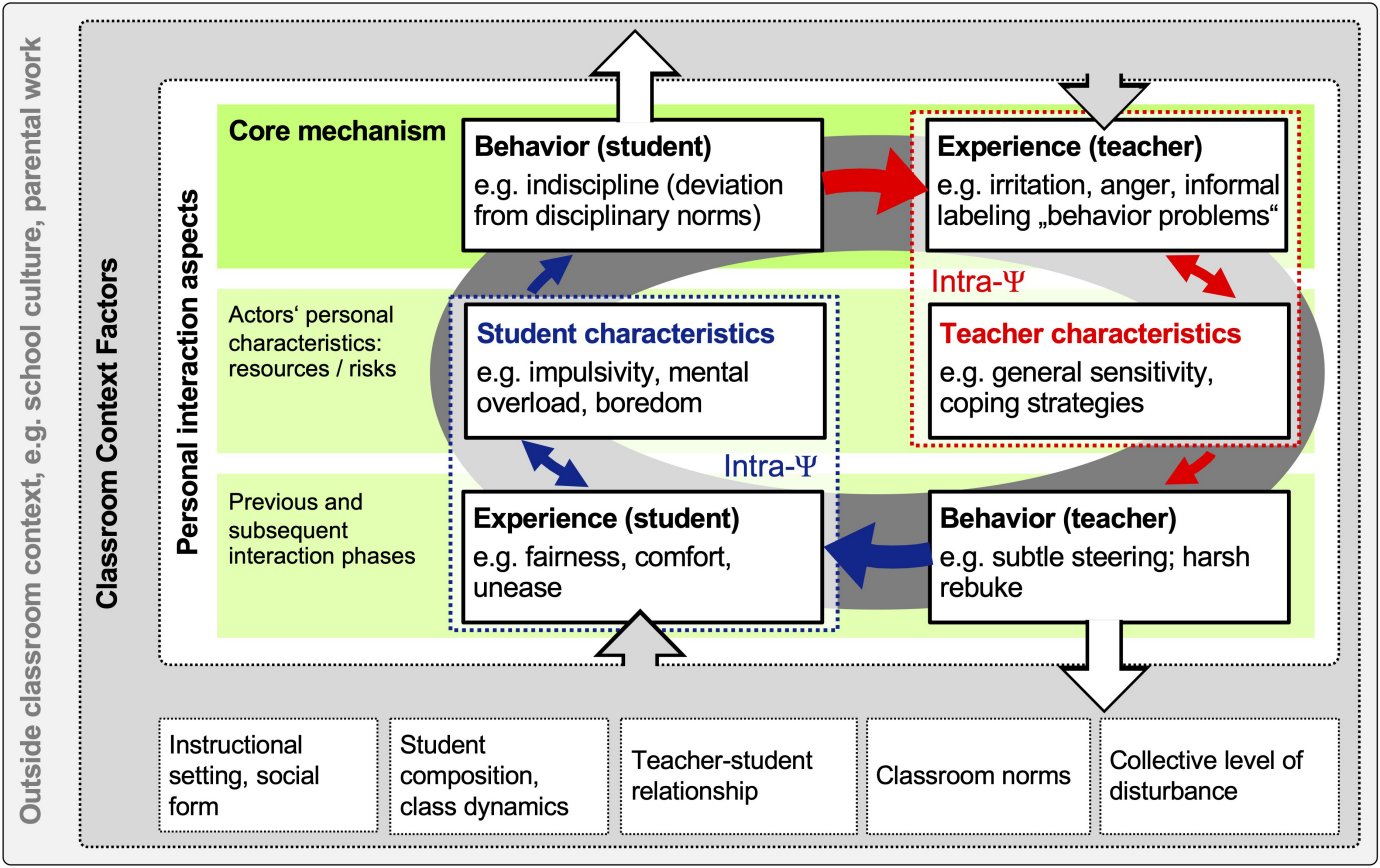
A theoretical process model of pedagogical interactions in the classroom.

Figure 1 shows these interrelated facets in a theoretical process model after Eckstein et al. (under review). It is a heuristic that attempts to illustrate the intertwined effects in high resolution and as holistically as possible. The top green bar represents the interactional core mechanism: an actor exhibits a behavior (exemplified as a student’s indiscipline) – which is experienced by an interaction partner (exemplified as the teacher’s irritation, anger, and labeling).

On the one hand, the student’s behavior is affected by his/her individual characteristics, e.g., impulse control (Carroll et al., 2006) or sex (Schwab et al., 2018). Besides, contextual factors can influence the student’s behavior, e.g., classroom organization (Godwin et al., 2016) or instructional clarity (Eckstein et al., 2022a).

On the other hand, the teacher’s experience of the student’s behavior represents an intrapsychic process. Unsurprisingly, this experience is strongly associated with the student’s actual behavior (e.g., severity of the indiscipline) (Eckstein, 2019). Beyond that, non-behavioral characteristics of this student can influence the teacher’s experience (e.g., learning ability) (Zevenbergen, 2001). Also, the teacher’s individual characteristics can affect his/her experience (e.g., general sensitivity to disturbances) (Marusic, 2023). Further, contextual factors can impact the teacher’s experience of the student’s behavior (e.g., the class’s collective level of indiscipline) (Hofstetter, 2022). If the teacher concludes that a critical threshold has been crossed, he/she may deduce that this student has behavior problems (Hempel-Jorgensen, 2009).

In general, the model illustrates a cyclic pattern: depending on how the teacher appraises the student’s behavior (e.g., as provocative or unintentional), he/she will react with a behavioral response (e.g., harsh rebuke or subtle prompt). This reaction is influenced by personal characteristics (e.g., work-related stress experience), professionally acquired competencies (e.g., pedagogical expertise), and contextual factors (e.g., current instructional setting) (Blömeke et al., 2015). As the interaction continues, the student will experience the teacher’s reaction as more or less appropriate and respond accordingly. Strack and Horowitz (2011) refer to this reciprocal relationship between the actors’ behaviors and experiences across interaction phases as “transactional causality” (p. 1). Finally, the model’s nested structure illustrates that pedagogical interactions occur within specific classroom contexts (Doyle, 2006; Müller and Hofmann, 2014).

Although Figure 1 appears complex due to the numerous illustrated processes, it simplifies reality: classroom teaching involves many sequential and sometimes simultaneous pedagogical interactions in a group setting with several students. The resulting multi-variant dynamics cannot be visualized graphically but can be imagined as several interwoven spirals (Eckstein et al., under review).

### 2.3 Interactionist considerations on labeling conditions

Based on interactionist theory and previous research, it can be considered to what extent students’ non-behavioral characteristics may affect teachers’ tendencies to label them as having behavior problems. Studies found that boys show deviant behaviors in class more often than girls (e.g., Schwab et al., 2018). Since most teachers presumably know this, a follow-up question arises from the interactionist perspective: To what extent does this knowledge lead to sex-role stereotypes and cause teachers to overestimate the problem among boys? Results from early gender-labeling research are inconsistent (Stern and Karraker, 1989). Indications can be drawn from more recent studies on effects that may bias teachers’ evaluation of student achievement. For example, Terrier (2020) identified systematic grading biases in favor of girls and against boys, with long-term consequences for the students’ school careers. Similarly, Ferman and Fontes (2022) demonstrated that many teachers tend to confuse students’ non-cognitive characteristics with their competence, resulting in inflated grades for well-behaved students and girls. Further evidence suggests that these mechanisms are intertwined: Contreras (2024) found that student behavior moderates teachers’ grading biases, i.e., discrimination decreases when boys exert greater learning effort. According to an interactionist interpretation of these findings, female sex and favorable learning ability may protect students from becoming labeled as having behavior problems by their teacher.

Beyond that, teachers’ personal characteristics can be considered possible conditions for their tendency to label students as having behavior problems. Teachers perceive student behavior through the lens of their habitual mental filters, resources, and risk factors. Wettstein et al. (2023) showed that teachers with a high resignation tendency and chronic worry systematically overestimate student aggression. Therefore, it can be assumed that teachers experiencing high levels of work-related stress are particularly likely to label their students as having behavior problems. Moreover, the teachers’ general sensitivity to disturbances is associated with the degree to which they perceive individual students as disturbing (Eckstein et al., 2022a). Thus, sensitivity can be assumed as a further condition of teachers’ tendency to label students as having behavior problems.

In addition, contextual factors can be considered conditions for teachers’ labeling tendencies. Makarova et al. (2014) found that the collective level of deviant behaviors in a class affects the teacher’s and classmates’ experience of individual students’ deviance: in tumultuous classes, individual deviance is perceived as less irritating than in tranquil classes. This finding can be interpreted in two ways. First, teachers and classmates feel particularly disturbed by individual deviance when the class as a collective forms a norm-compliant contrast (reference group effect). Second, teachers and students get accustomed to deviant behaviors in tumultuous classes, which desensitizes them over time (habituation effect). It can therefore be assumed that students in classes with low collective levels of deviant behaviors are particularly likely to be labeled as having behavior problems by their teachers. Furthermore, instructional clarity can be considered a possible labeling condition. This is because unclear instructions may prompt several students to request assistance simultaneously, while others may engage in off-task behavior. Such multiple demands present a significant challenge to teachers, which most experience as stressful (Kokkinos, 2007). As a result of this stress, teachers may be more inclined to label students as having behavior problems.

Finally, these interactionist considerations imply methodological challenges for empirical studies (Eckstein, 2018). A key issue is that student behavior problems cannot be investigated objectively from a single perspective. This is because the phenomenon is perspective-dependent and human perception is susceptible to biases (Hoyt, 2000; Wolfe, 2004). To approximate objectivity, researchers may assess multiple perspectives and estimate an intersubjectively shared view (Eckstein, 2019). Ideally, this includes an external perspective, e.g., systematic observation (Pianta and Hamre, 2009). If observation is unavailable, ratings of multiple students from the same classes are a suitable alternative, since individual under- and overestimates cancel out in aggregation. As a result, aggregated student ratings usually correlate more strongly with external observations than teacher ratings do (Den Brok et al., 2006; Montuoro and Lewis, 2015; Scherzinger and Wettstein, 2019).

In sum, we need more empirical evidence on the assumed effects of non-behavioral student characteristics, teacher characteristics, and contextual factors on teachers’ tendencies to label students as having behavior problems. To this end, research ideally takes into account methodological implications derived from interactionist considerations. This paper presents a study that contributes to this aim.

## 3 Research question and hypotheses

Based on previous findings and interactionist reasoning, we postulate two fundamental assumptions concerning two main types of labeling conditions:

- Teachers primarily label students as having behavior problems if they exhibit deviant behaviors with comparatively high frequency or severity. In other words, teachers do not label students arbitrarily or without reason.
- Teachers’ labeling of students as having behavior problems may be biased, as their perceptions are affected by students’ non-behavioral characteristics, teacher characteristics, and contextual factors.

The current state of research does not provide sufficient knowledge about the extent to which teachers’ labeling is substantiated by students’ behaviors (assumption 1) – and the extent to which it is due to other, more idiosyncratic conditions (assumption 2). For practice, however, it would be essential to understand the impact of these two condition types, as they require different problem-solving strategies. Addressing this gap, we investigated the following research question:

*Under which conditions do teachers label students as having behavior problems?*

We examined potential conditions by testing seven hypotheses. The first hypothesis corresponds to assumption 1, which is supported by empirical findings on teacher-appraised severity of deviant student behaviors (e.g., Beaman et al., 2007):

- H1 – indiscipline: The more often an individual student shows undisciplined behaviors, the more likely the teacher will label this student as having behavior problems.

The other six hypotheses correspond to assumption 2, suggesting additional labeling conditions independent of the indiscipline effect assumed by H1.

*Non-behavioral student characteristics*

Research on the accuracy of teachers’ performance evaluation suggests biases resulting from students’ female sex (e.g., Terrier, 2020) and favorable learning disposition (e.g., Stang and Urhahne, 2016). Applying these findings to teachers’ appraisal of student behavior problems, we hypothesize:

- H2 – sex: Girls are less likely to become labeled as having behavior problems than boys.
- H3 – subjective experience of instructional clarity: The clearer an individual student experiences the teacher’s instruction, the less likely this student is to become labeled as having behavior problems.

*Teacher characteristics*

Studies on teacher-appraised student behavior problems suggest biases resulting from stress (e.g., Wettstein et al., 2023) and sensitivity (e.g., Eckstein et al., 2022a). Based on these findings, we hypothesize:

- H4 – work-related stress experience: The more stressful a teacher experiences his/her job, the more likely he/she will label students as having behavior problems.
- H5 – sensitivity: The more sensitive a teacher is to disturbances in general, the more likely he/she is to label students as having behavior problems.

*Contextual factors*

We are aware of only limited empirical research on contextual factors that may affect teachers’ appraisal of student behavior problems. Regarding the collective level of student deviance in classes, we rely on Makarova et al. (2014) who found that teachers are less irritated by individual deviance in tumultuous classes than in tranquil classes. As regards pedagogical aspects, we extrapolate from findings of related studies: Instructional clarity can facilitate students’ engagement in the lessons, which may protect teachers from overextension (e.g., Skaalvik and Skaalvik, 2016) and, thus, mitigate their appraisal of student behavior problems. Thus, we assume the following context effects:

- H6 – collective level of indiscipline: The more indiscipline the students in a class show collectively, the less likely their teacher is to label students as having behavior problems.
- H7 – average experience of instructional clarity: The clearer the students in a class experience the instruction on average, the less likely their teacher is to label them as having behavior problems.

## 4 Materials and methods

### 4.1 Participants, sampling, and procedure

This analysis is part of the SUGUS study, financed by the Swiss National Science Foundation (grant number: 100019_152722). The project was reviewed and approved by the Ethics Committee of the University of Zurich. Participation in the study was voluntary. Before recruitment, we asked local authorities for permission to access the field. If permission was granted, we asked principals to recommend the teachers at their school to participate in the study. Teachers who decided to enroll provided written consent after being informed of the study’s aims and procedures, as well as their rights as participants. Likewise, students and their parents were asked for written consent.

Data collection took place in the summer of 2016. The sample comprised 85 elementary school classes (90% fifth grade, 10% mixed grades; students’ mean age: 11.7 years, SD: 0.5 years). All 85 class teachers and 1,412 students out of 1,687 answered a survey during class time; 275 students did not answer the survey because they chose not to participate or because their parents did not allow it. The survey was administered at two time points 1 week apart (t1, t2), constituting a short-term longitudinal design with a predominantly cross-sectional character.

The questionnaires comprised specific sections on individual target students. Teachers reported on all students in the class (teacher ratings), students reported on four randomly assigned classmates (peer ratings), and themselves (self-ratings). At t2, all respondents stated whether they considered the targets to have behavior problems; this paper focuses on the teachers’ responses, interpreted as their labeling tendencies. One week before (t1), all respondents rated the frequency of the targets’ undisciplined behaviors as a presumed key condition of teachers’ labeling tendencies; the analysis in this paper relies on the peer ratings. The 1,412 students who responded to the survey served as both raters and targets (active participation), whereas the 275 non-respondents were rated only by their teachers (passive participation). Raters and targets were paired using personalized questionnaires. Several measures were taken to guarantee the respondents’ anonymity. Supervising project team members reported that all students had been in a good mood after the survey.

Further, we gathered information on the presumed additional labeling conditions in generic questionnaire sections and from administrative data (i.e., non-behavioral target characteristics, teacher characteristics, contextual factors).

Detailed information on the research design can be found in the study’s technical report (Eckstein et al., 2018) and other contributions by Eckstein (2018) 2019. These publications, as well as all data and research materials (Eckstein et al., 2022b), are freely available via open access (cf. DOI under References).

### 4.2 Measures

The measures were developed in-house, partly based on existing instruments. All sources, scaling procedures, and common psychometric properties are documented in the technical report (Eckstein et al., 2018). In short: principal component analyses substantiated the assumed one-dimensionality of the constructs; scale analyses yielded acceptable to good internal consistency and item discrimination; descriptive statistics revealed the variables’ distributions, which, in case of students’ indiscipline and teachers’ labeling tendencies are right-skewed, as expected, suggesting that only few students frequently showed indiscipline and were decidedly labeled as having behavior problems.

Moreover, Eckstein (2018, 2019) investigated the consistency across teacher, peer, and self-ratings of the target-specific measures. Analyses of variance and a two-level correlated trait – correlated method minus one model yielded extensive results. In short, consistency coefficients amounted to 7%–54% (interrater agreement), while rater-specific divergences amounted to 30%–70% (rater effects). As an exemplary mean difference, teachers reported the highest incidence rates of indiscipline compared to peer and self-ratings (F[1.49, 2046.56] = 76.08, *p* < 0.001, η^2^ = 0.05).

The analyses in this paper were based on two single items and four multi-item constructs (modeled by item parcels); the example items below are English paraphrases of the original German wording:

- Labeling: The teachers reported the degree to which they subjectively considered the target students to have behavior problems by a single item: “In my opinion, student X has behavior problems.” They answered this item individually for each student in their class using a four-point response format (0 = strongly disagree … 3 = strongly agree).
- Indiscipline: Four classmates were randomly assigned to rate the frequency with which the target students showed undisciplined behaviors on a low-inference scale. On average, 3.47 peer ratings per target were collected. The scale comprised eight items, e.g., “Student X made noise in class.” The peers reported the number of occurrences in the past 2 weeks using a six-category response format (“Never” to “5 times”) and an option for free responses (“More frequently, namely: …”). The interrater agreement per target across peers amounts to 31% (Eckstein, 2019). The four peer ratings per item and target were aggregated into manifest mean scores and bundled into three parcels (McDonald’s Omega = 0.84).
- Sex: The target students’ sex was extracted from administrative records. It was coded dichotomously (0 = boy, 1 = girl).
- Instructional clarity: The target students reported how clear and understandable they found the teacher’s instruction. The scale comprised six items, e.g., “When the teacher explains something, I always understand it.” The students answered these items using a four-point response format (0 = strongly disagree … 3 = strongly agree). The items were bundled into three parcels (McDonald’s Omega = 0.72).
- General sensitivity: The teachers reported their general sensitivity to classroom disturbances by rating 16 prototypical incidents, e.g., “Two students box each other under their desks in class.” Using a four-point response format, they appraised the degree of disturbance they generally perceive in such situations (0 = not at all disturbed … 3 = strongly disturbed). The 16 items were bundled into three parcels (McDonald’s Omega = 0.71).
- Stress experience: The teachers reported their work-related stress experience on a scale comprising four items, e.g., “My job is too strenuous for me,” using a four-point response format (0 = strongly disagree … 3 = strongly agree). The four items were bundled into two parcels (McDonald’s Omega = 0.80).

### 4.3 Analysis strategy

A two-level structural equation model (SEM) was set up and analyzed in Mplus 8.10 (Muthén and Muthén, 2017–2023). The model estimated the relationships between teachers’ labeling tendencies and three covariates at the student level (L1) and four covariates at the teacher/class level (L2). All effects were estimated while controlling for the others. Furthermore, all covariates were allowed to correlate. The two single items (labeling, sex) were modeled as stand-alone manifest indicators, and the four constructs (indiscipline, instructional clarity, general sensitivity, stress experience) as latent factors based on multiple item parcels.

Given the nested data structure (19.8 students were clustered in 85 classes/teachers on average in the analysis sample), level-specific conditions of the teachers’ labeling were investigated. This multilevel approach was also indicated to calculate the standard errors adequately (Stapleton et al., 2016). The original L2 variables (general sensitivity, stress experience) were modeled as latent factors at the teacher/class level. The original L1 variable sex was modeled exclusively at the student level. The other three original L1 variables (labeling, indiscipline, instructional clarity) were modeled at both levels, with the L2 indicators being estimated as latent random intercepts (Marsh et al., 2009). This modeling technique corresponds to latent group-mean centering, implying an orthogonal decomposition of variance components at levels 1 and 2 (with values at L2 representing latent class means and values at L1 representing individual deviations from the class means). As a consequence, the three variables have level-specific meanings:

- Labeling: At L2, the aggregated variable reflects a teacher’s average tendency to label the students in the class as having behavior problems, i.e., this teacher’s general labeling tendency across students. At L1, the variable reflects a teacher’s tendency to label an individual student relative to his/her general labeling tendency.
- Indiscipline: At L2, the aggregated variable reflects the average frequency with which the students of a class showed undisciplined behaviors, i.e., the collective level of indiscipline in a class. At L1, the variable reflects how frequently an individual student showed undisciplined behaviors relative to his/her class’s collective level of indiscipline.
- Instructional clarity: At L2, the aggregated variable reflects the intersubjectively shared experiences of all students in a class regarding instructional clarity, i.e., their common view of instructional clarity. At L1, the variable reflects those elements of an individual student’s subjective experience of instructional clarity not shared with classmates, e.g., due to his/her specific learning ability.

Beyond that, indiscipline and instructional clarity were modeled with metric measurement invariance between the two levels to avoid cluster bias: the factors’ unstandardized loadings were set equal between L1 and L2 (Jak et al., 2013). Further, the measurement errors were assumed to be entirely located at L1. Therefore, the residual variances of the L2 factor indicators were fixed to zero, yielding standardized L2 factor loadings of 1.00 (Eid et al., 2008).

Additional measures were adopted: considering the data’s ordinal character and non-normality, the WLSMV estimator (weighted least squares mean- and variance-adjusted) and Mplus-specific CATEGORICAL option were applied as recommended by Finney and DiStefano (2013). Because the number of parameters threatened to become very large and thus thwart estimation, the original items were clustered into parcels by calculating manifest mean scores of multiple item scores in advance. To still be able to use the CATEGORICAL option, the parcel decimal values were rounded to integers. Missing values were treated with the Mplus default setting of estimating models using all available information (Asparouhov and Muthén, 2010). This meant, in particular, that the data of passively participating students could also be used for the estimation, although they only comprised teacher ratings (no self- and peer ratings).

## 5 Results

The global and the level-specific model fit indices (Ryu and West, 2009) suggest that the estimation was broadly consistent with the data (global: χ^2^ = 139.468, df = 71, *p* < 0.001; RMSEA = 0.024; CFI = 0.991; L1: χ^2^ = 83.31, df = 16, *p* < 0.001; RMSEA = 0.051; CFI = 0.993; L2: χ^2^ = 53.63, df = 45, *p* = 0.177; RMSEA = 0.048; CFI = 0.896). Given the rules of thumb suggested by Hu and Bentler (1998), the CFI could indicate a misfit at L2, whereas all other indices suggest an acceptable to good fit. Altogether, we concluded that the model fit suffices. Furthermore, the overall results indicate that the model was specified adequately. For example, all factorial structures yielded expected results, with most standardized factor loadings greater than 0.7 and none less than 0.6.

**FIGURE 2:**
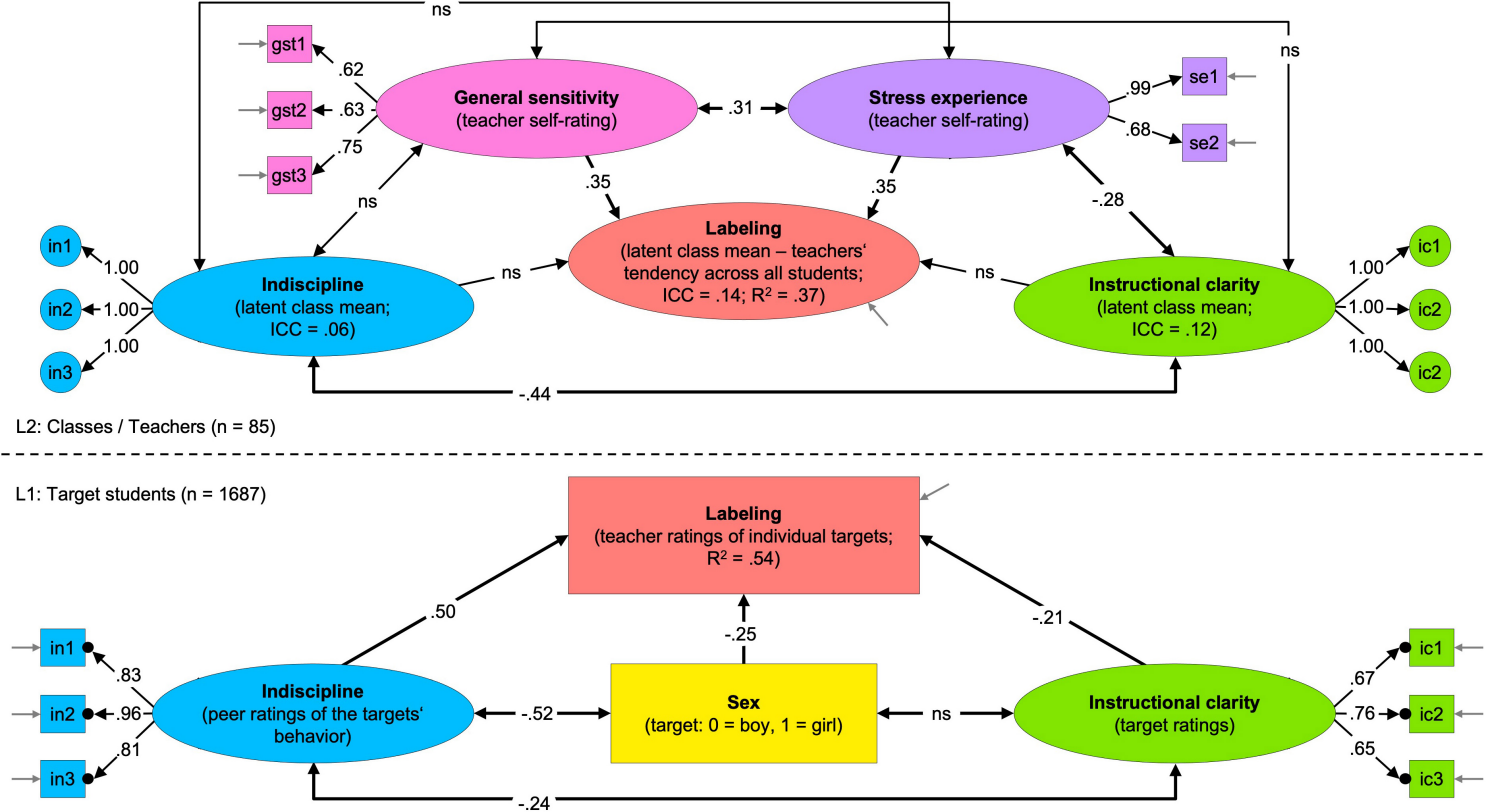
Sketch of the results of the two-level structural equation model (SEM) “labeling conditions.” Ovals symbolize latent factors; boxes represent manifest indicators; one-headed arrows illustrate factor loadings or regression slopes; double-headed arrows represent correlations; short gray arrows without origin symbolize measurement errors. The lower part of the figure represents L1, and the upper part is L2. The black dots on the edge of the L1 indicators correspond to the L2 circles, symbolizing that the L1 indicators were aggregated into error-free random intercepts at L2. All numbers depicted in the sketch refer to significant standardized estimates (*p* < 0.05); non-significant parameters are denoted with “ns.”

Figure 2 shows a sketch of the model, including essential results, following the conventions suggested by Muthén and Muthén (2017–2023). At the model’s core is the teachers’ labeling. This variable’s intraclass correlation coefficient (ICC[1]) indicates that 14% of the variance was at L2, reflecting meaningful differences in teachers’ general tendencies to label students as having behavior problems. The remaining 86% of the variance was at L1, reflecting differences in the teachers’ view of individual students. This finding justifies our investigation of (i) teacher characteristics and context factors as explanatory sources of L2-variance, (ii) behavioral and non-behavioral student characteristics as explanatory sources of L1-variance.

The ICC(1) for indiscipline indicates that 6% of its variance was at L2, revealing that the collective level of indiscipline varied (a little) across classes, whereas 94% of its variance reflects interindividual differences. Further, the ICC(1) for instructional clarity indicates that 12% of its variance was at L2, revealing (some) between-class differences, whereas 88% of its variance reflects interindividual differences.

At the student level (L1, *n* = 1687), the largest effect was found for indiscipline (β = 0.50, *p* < 0.001), indicating, as expected, an increasing labeling tendency for individual students who showed undisciplined behaviors with increasing frequency. Since all effects were estimated while controlling for the others, any further significant coefficient can be interpreted as an effect independent of students’ indiscipline. For the target students’ sex, a negative effect on teachers’ labeling was found (β = −0.25, *p* < 0.001), as expected, indicating that girls were less likely to become labeled as having behavior problems than boys – regardless of their indiscipline frequency. Similarly, a negative effect on labeling was found for the targets’ subjective experience of instructional clarity (β = −0.21, *p* < 0.001). This suggests, in line with the expectations, that students were less likely to become labeled the clearer they experienced the instruction – regardless of their indiscipline frequency. Negative correlations between indiscipline and sex (*r* = −0.52, *p* < 0.001) and indiscipline and instructional clarity (*r* = −0.24, *p* < 0.001) indicate that girls and students with subjective experiences of high instructional clarity showed indiscipline comparatively rarely. In sum, the proportion of variance explained for the teachers’ labeling of individual students (R^2^ L1) amounts to 54.0% (*p* < 0.001).

At the teacher/class level (L2, *n* = 85), no significant effect was found for the collective level of indiscipline (β = −0.05, *p* = 0.844) or instructional clarity (β = −0.12, *p* = 0.561). This suggests that the teachers’ labeling tendencies across students were not associated with these contextual factors, contrary to our expectations. However, a negative correlation between these two variables was found (*r* = −0.44, *p* = 0.013), indicating that higher levels of instructional clarity were associated with lower levels of collective indiscipline and vice versa. Further, positive effects of teacher characteristics were found: the more sensitive teachers were to disturbances (β = 0.35, *p* = 0.011) and the more stressed they felt in their job in general (β = 0.35, *p* = 0.014), the more likely they were to label students as having behavior problems – regardless of the students’ indiscipline frequency. In addition, these two constructs correlate significantly (*r* = 0.31, *p* = 0.014), indicating that more sensitive teachers felt more stressed and vice versa. Beyond that, a negative correlation between stress experience and instructional clarity was found (*r* = −0.28, *p* = 0.023), indicating that higher teacher stress was associated with lower instructional clarity. In sum, the proportion of variance explained for the teachers’ labeling tendencies across all students in their class (R^2^ L2) amounts to 36.5% (*p* = 0.016).

## 6 Discussion

### 6.1 Summary and interpretation of the findings

This paper investigated conditions under which teachers informally label students as having behavior problems. We examined two fundamental assumptions, relying on an interactionist framework (Eckstein et al., under review). Based on survey data from 85 elementary school classes, we tested seven hypotheses with a two-level SEM.

The results support the first fundamental assumption corresponding to hypothesis 1 (H1 accepted): teachers’ tendencies to label individual students as having behavior problems increased when the frequency of the individual students’ indiscipline increased. This effect alone explains about 25% of the variance in labeling, a substantial effect size.

According to the second fundamental assumption, students’ non-behavioral characteristics, teacher characteristics, and context factors have additional explanatory power. We investigated this second assumption by testing six further hypotheses (H2–H7). Each effect was estimated while controlling for all other effects, which means in particular that any confounding with indiscipline is accounted for and neutralized. In other words, all other effects hold regardless of how often the students showed indiscipline.

Both non-behavioral student characteristics were found to have significant effects on the teachers’ labeling tendencies. First, teachers were less inclined to label girls as having behavior problems than boys (H2 accepted). This finding can be interpreted by considering previous research on the impact of sex-role stereotypes (Imhoff and Hoffmann, 2023): since the notion of behavior problems fits better with male than female stereotypes, teachers may have been inhibited from labeling girls or reinforced in labeling boys. Second, the clearer students experienced their teacher’s instruction, the lower was the teacher’s tendency to label them as having behavior problems (H3 accepted). Two interpretations are conceivable: (i) Students with favorable learning ability (e.g., highly developed language skills) may have been able to talk their way out of emerging conflicts with the teacher, while students with less favorable learning ability reacted more clumsily in such situations and thus ran a greater risk of becoming labeled. (ii) Teachers may have overestimated behavior problems among low-achieving students or underestimated them in high-achievers. This bias may have arisen from confusion between students’ learning and disciplinary behavior – due to a halo effect (Stang and Urhahne, 2016) or stereotypical beliefs about students that are “easily distracted and disruptive” (Anderson et al., 2012, p. 514).

Beyond that, both teacher characteristics were found to have significant effects: First, the more stressful teachers experienced their job, the more likely they were to label students as having behavior problems (H4 accepted). Second, an increase in teachers’ general sensitivity to classroom disturbances was associated with stronger tendencies to label students (H5 accepted). These findings support the assumption that personal characteristics affect how teachers experience student behavior and, thus, the degree to which they are inclined to assign the behavior-problem label (Eckstein et al., 2022a; Wettstein et al., 2023).

Contrary to expectations, no evidence was found for the assumed context effects. First, the classes’ collective levels of indiscipline were not significantly associated with the teachers’ labeling tendencies (H6 rejected). A possible explanation is that two opposing mechanisms exist: (i) In tumultuous classes, teachers’ labeling tendencies may decrease over time because they normalize deviant student behavior due to a reference group or habituation effect (initially expected after Makarova et al., 2014). (ii) In tumultuous classes, teachers’ labeling tendencies may increase over time due to a transfer or accumulation effect – the stress caused by the collective promotes the labeling of individuals. If both mechanisms act simultaneously, they will neutralize each other with a net effect of zero. Further, no significant effect was found for instructional clarity at the teacher/class level (H7 rejected). We assumed the following pathway: when students experience the instruction as clear, they can easily engage in the lessons, which protects their teachers from overextension (Epel et al., 2018). As an indirect consequence, teachers’ labeling tendencies would be reduced. Although the results do not support this assumption, we hesitate to discard the theoretically plausible mechanism. One possible explanation is that uncontrolled effects confounded the results. For example, teachers with reduced flexibility and openness may simultaneously tend toward heightened labeling tendencies and toward direct instruction, which many students appraise as clear (Hugener et al., 2009). This confounding would obscure the theoretically assumed protective effect of instructional clarity on labeling. An alternative explanation for the null effect refers to the methods applied: they might not have been sophisticated enough to trace the assumed pathway empirically.

### 6.2 Strengths and limitations

Many previous studies have relied on pathogenetic approaches to examine student behavior problems, focusing narrowly on the students’ behaviors and their causes. Other studies focused on teachers’ experience and labeling of student behavior problems, relying on perceptual psychology or the labeling approach. Some of these studies were built exclusively on one approach while ignoring others. In contrast, a strength of our study lies in its interactionist approach that investigates both the students’ behaviors and the teachers’ (idiosyncratic) experience and labeling (Eckstein et al., 2016).

Further strengths of the SUGUS study lie in its methodological design. We collected ratings of individual targets from a sample of 1,412 actively participating students. This allowed for analyses of interactional processes with comparatively high resolution (higher than in conventional surveys without target-specific ratings) and generalizability (higher than in case studies with small sample size). In addition, the multi-informant approach enabled analysis of the relationships among the variables of interest from multiple perspectives, e.g., peer ratings for behavior assessment, teacher reports on informal labeling, and target student ratings of instructional quality. This broad basis for the analyses strengthens the validity of the results. Furthermore, the multiple peer ratings of the individual target students’ behaviors can be viewed as a strength as they approximate an objective measure (better than self- or teacher ratings).

The SUGUS study also has limitations. For one thing, it lacks observational data that might even better approximate an objective measure of student behaviors than peer ratings. Besides, the focus on individual students’ behaviors limited the capability to investigate contextual factors with the same rigor. For example, instructional practices were captured by retrospective teacher and student ratings that were rather general and may not have been tangible enough.

Other limitations arise from the study’s temporal design and cross-sectional character. (i) Given the short time lag between data collection points, the conditional effects tested in the statistical model are, in fact, rather correlational. (ii) Since teachers and students interact in reality for longer periods than those investigated, it remains unclear whether the observed associations validly represent true effects or constitute over- or underestimates due to short-term dynamics. (iii) The two core variables refer to different time scales. While the low-inference peer ratings of target students’ indiscipline explicitly covered the 2 weeks preceding data collection, teachers’ labeling was not bound to a specific time frame. Thus, teachers likely reflected a longer time horizon when reporting the degree to which they considered the targets to have behavior problems.

Further, the sample quality is unclear. Since participation was voluntary, no random sample could be obtained. Therefore, the findings’ representativity is uncertain. For example, the estimated relationships between teachers’ personal characteristics and labeling tendencies could be biased if specific teacher-student combinations were over- or underrepresented. Rather unlikely are systematic biases arising from administrative teacher-student matching, since class assignment is not coherently standardized in Switzerland. Yet, self-selection bias is conceivable: possibly, teachers with comparatively low work-related stress experiences enrolled predominantly, resulting in artificially low variance and, thus, reduced explanatory power of our analysis.

Finally, the parceling of indicators may be considered less than ideal. Bandalos (2002) notes that parceling can obscure misfit or bias estimates in the case of multidimensional constructs. Yet Little et al. (2002) consider parceling acceptable in the case of unidimensional constructs and when analyses focus on inter-construct relations rather than internal factor structures – both of which apply to our study. Furthermore, preliminary analyses, including logistic regression and multilevel analysis with the MLR estimator, yielded results that are consistent with those presented here (so far not published in text form but presented at conferences, cf. Eckstein et al., 2023), which speaks for the overall stability of the findings.

### 6.3 Scientific relevance

The results broadly support the theoretically established assumptions: teachers’ labeling of students as having behavior problems can be understood as an interactionist process influenced by the students’ behavior and several non-behavioral conditions. As expected, students’ undisciplined behavior was found to be a dominant condition of the teachers’ labeling. In addition, students’ sex, subjective experience of instructional clarity, and teachers’ sensitivity to disturbances and work-related stress experience were identified as significant conditions. These findings imply, to some degree, perception biases and emphasize the partly idiosyncratic character of teachers’ labeling of students as having behavior problems. They elaborate on the current state of research and can serve as a point of reference for future studies that may deepen understanding of the conditions under which students are considered to have behavior problems.

Having shown that it is productive to investigate students’ classroom behaviors and teachers’ behavior perceptions simultaneously with equal rigor, we recommend that future studies do the same. We suggest an interactionist theoretical framework to conceptualize such holistic approaches (Eckstein et al., under review). Ideally, research should take into account methodological issues emerging from the problem’s multi-faceted, perspective-dependent character (Eckstein, 2018), for example, by adequately operationalizing the students’ disciplinary and social behavior (Eckstein, 2019), combining survey methods with systematic observation (Pianta and Hamre, 2009; Wettstein et al., 2023), or longitudinal designs with sufficient time lag between data collection points (Jenni et al., 2025). In addition, future studies may examine other conditions affecting teachers’ labeling beyond those we investigated. For instance, students’ socio-cultural background may have similar effects as sex on both students’ actual behaviors and teachers’ labeling tendencies (Zevenbergen, 2001). Furthermore, qualitative methods may extend the existing knowledge on the impact of teacher characteristics (e.g., pedagogical attitude) and contextual factors (e.g., instructional setting) on teachers’ experience of student behaviors (Hofstetter, 2022; Marusic, 2023).

### 6.4 Implications for practice

In line with interactionist theory and previous research, the present study substantiates that teacher-assigned student behavior problems comprise (i) student-related behavioral aspects and (ii) teacher-related perceptual aspects. To mitigate these aspects through targeted interventions, teaching practice requires different problem-solving strategies.

First, behavioral aspects require pedagogical interventions. Teachers must co-regulate deviant student behaviors, e.g., by classroom management (Emmer and Sabornie, 2015) or emotional support (Gasser et al., 2018). In addition, special interventions such as Banking Time (Williford and Pianta, 2020) can help teachers connect with students who would otherwise find it difficult to accept co-regulation. Training courses on such pedagogical interventions may empower teachers to prevent and effectively deal with deviant student behavior (Wettstein and Scherzinger, 2022). Furthermore, such trainings may have beneficial effects that go beyond behavior management: when teachers feel less stressed due to enhanced agency in dealing with deviant behaviors, their risk of overestimating the problem may be mitigated as well (Kyriacou et al., 2007; Kunz Heim et al., 2019).

Second, perceptual aspects require teacher-centered interventions, especially when non-behavioral conditions bias teachers’ labeling. Since the label “behavior problems” can have drastic consequences for students (Bernburg, 2019), teachers must be sensitized to how blind spots or prejudices can result in over- or underestimation. A promising approach might be to promote mentalizing strategies that help teachers better understand students’ rationales for exhibiting deviant behavior. In general terms, student deviance can be seen as a symptom of overextension due to a lack of cognitive, motivational, or socio-emotional resources required to comply with behavioral expectations in class (Fischer, 1995). Raising teachers’ awareness of such mechanisms in training courses may help them effectively deal with (stressful) student deviance. In line with this assumption, Kumschick et al. (2018) showed that teachers who know a student’s perspective for his/her deviant behavior are more likely to apply functional emotion regulation strategies compared to teachers who do not know the student’s perspective.

A further implication for practice concerns public discourse on the inclusion of students with behavior problems: many contributions to this discourse locate the problem one-sidedly in students, sometimes combined with the demand to exclude these “troublemakers.” Other statements locate the problems one-sidedly in teachers, sometimes insinuating a lack of competence or ill intent. Such personalizing, reproachful contributions are symptoms of an agitated atmosphere with many disadvantages (Bernhardt, 2007). It would greatly benefit teachers and students if the discourse were less agitated and more differentiated. We hope to have made a small contribution in this direction with our paper.

## Data Availability

The datasets presented in this study can be found in online repositories. The names of the repository/repositories and accession number(s) can be found below: SWISSUbase repository: https://doi.org/10.48573/w1q3-kt72.
