# Correction: Teachers' labeling of student behavior problems: a multiperspective study of teacher, student, and classroom conditions

**DOI:** 10.3389/fpsyg.2026.1854525

**Published:** 2026-05-07

**Authors:** Frontiers Production Office

**Affiliations:** Frontiers Media SA, Lausanne, Switzerland

**Keywords:** behavior problems, interactionism, labeling approach, multi-informant design, perception bias, structural equation modeling

Figures 1 and 2 were in the wrong order in the PDF/HTML versions of this paper. The image currently shown as Figure 1 should be Figure 2, and vice versa. The order has now been corrected.

**Figure 1:**
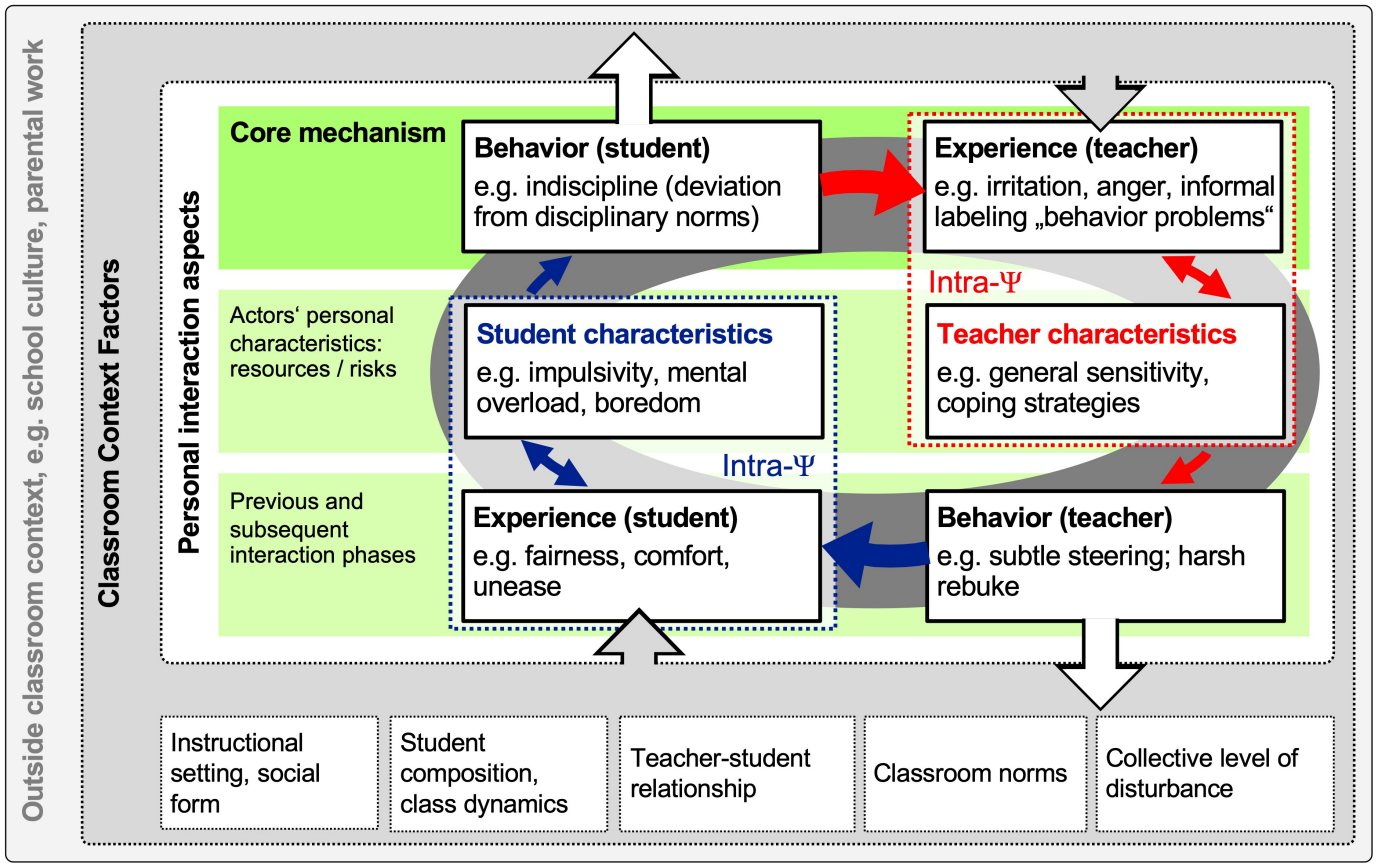
A theoretical process model of pedagogical interactions in the classroom.

**Figure 2:**
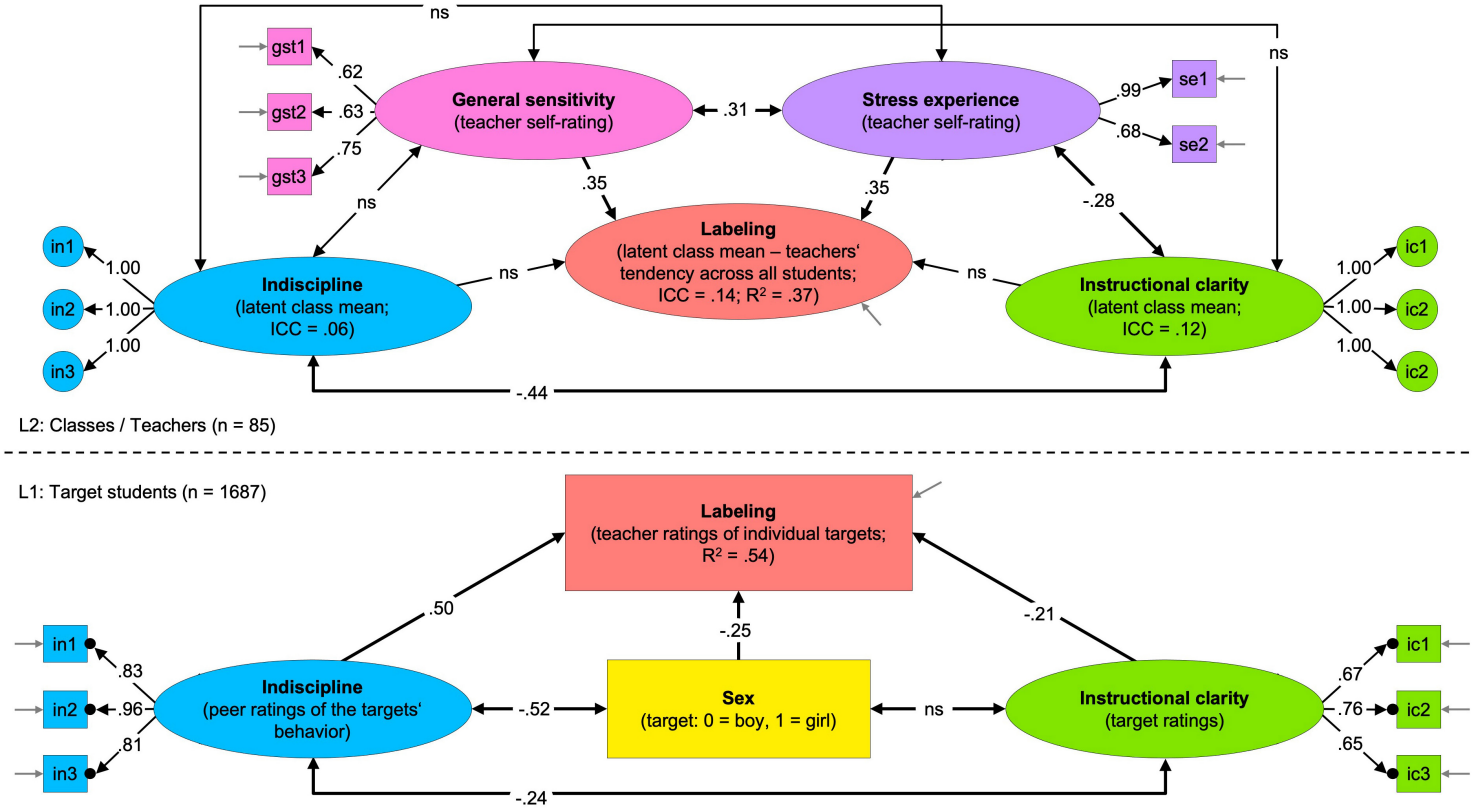
Sketch of the results of the two-level structural equation model (SEM) “labeling conditions.” Ovals symbolize latent factors; boxes represent manifest indicators; one-headed arrows illustrate factor loadings or regression slopes; double-headed arrows represent correlations; short gray arrows without origin symbolize measurement errors. The lower part of the figure represents L1, and the upper part is L2. The black dots on the edge of the L1 indicators correspond to the L2 circles, symbolizing that the L1 indicators were aggregated into error-free random intercepts at L2. All numbers depicted in the sketch refer to significant standardized estimates (*p* < 0.05); non-significant parameters are denoted with “ns.”

The original version of this article has been updated.

## Generative AI statement

Any alternative text (alt text) provided alongside figures in this article has been generated by Frontiers with the support of artificial intelligence and reasonable efforts have been made to ensure accuracy, including review by the authors wherever possible. If you identify any issues, please contact us.

